# Barriers and enablers of physical activity engagement for patients with COPD in primary care

**DOI:** 10.2147/COPD.S119806

**Published:** 2017-03-28

**Authors:** Maria-Christina Kosteli, Nicola R Heneghan, Carolyn Roskell, Sarah E Williams, Peymane Adab, Andrew P Dickens, Alexandra Enocson, David A Fitzmaurice, Kate Jolly, Rachel Jordan, Sheila Greenfield, Jennifer Cumming

**Affiliations:** 1 School of Sport, Exercise and Rehabilitation Sciences; 2 Institute of Applied Health Research, University of Birmingham, Edgbaston, Birmingham, UK

**Keywords:** COPD, social cognitive theory, self-efficacy, barriers, enablers, primary care

## Abstract

**Background:**

Given that physical activity (PA) has a positive impact on COPD symptoms and prognosis, this study examined the factors that both encourage and limit participation in PA for individuals with COPD in a primary care setting from the perspective of social cognitive theory.

**Methods:**

A purposive sample of 26 individuals with a range of COPD severity (age range: 50–89 years; males =15) were recruited from primary care to participate in one of four focus groups. Thematic analysis was undertaken to identify key concepts related to their self-efficacy beliefs.

**Results:**

Several barriers and enablers closely related to self-efficacy beliefs and symptom severity were identified. The main barriers were health related (fatigue, mobility problems, breathing issues caused by the weather), psychological (embarrassment, fear, frustration/disappointment), attitudinal (feeling in control of their condition, PA perception, older age perception), and motivational. The main enabling factors were related to motivation (autonomous or controlled), attitudes, self-regulation, and performance accomplishments.

**Clinical implications:**

When designing interventions for individuals with COPD, it is important to understand the patient-specific social cognitive influences on PA participation. This information can then inform individually tailored management planning.

## Introduction

COPD is a debilitating respiratory disease most commonly found in chronic smokers,^1^ which leads to both physical functional limitations (including increased breathlessness) and psychological distress (including anxiety and depression).^2^ There is a wide spectrum of disease, ranging from mild, where impact on physical functioning is minimal, to very severe, where patients benefit from end-of-life care. Underdiagnosis of COPD, particularly at the earlier stages, is widespread, with an estimated two-thirds of patients being undiagnosed in the UK.^3^

At all stages, engaging in physical activity (PA) may afford a variety of general and disease-specific health benefits, including reduced risk of hospitalization and death;^4^ thus, participation in daily PA is recommended by experts.^5^,^6^ Structured and supported PA is also an important component of the pulmonary rehabilitation (PR) programs recommended to individuals with COPD^7^ and has been shown to lead to clinical benefits, eg, decreased perception of dyspnea, improved muscle function, and enhanced exercise capacity.^8^ However, the majority of individuals with COPD are significantly less active on a daily basis than healthy older adults.^9^ As COPD symptoms become more severe, with decline in lung function and increased breathlessness, PA levels decline further.^10^ Inactivity can in turn lead to further deterioration of physical health, which is a further barrier to PA engagement.^11^

Previous studies have attempted to understand barriers and facilitators to engagement with PA among healthy older adults.^12^ Health issues,^13^ pain, weather, lack of time,^14^ lack of transportation, and fatigue^15^ are the most commonly cited barriers, while enablers include physical, psychological, social, and environmental motivators.^16^ In addition to the general barriers, individuals with COPD report disease-specific problems, including breathlessness and negative experiences with PR programs,^17^ as well as limitations arising from comorbidities, feelings of shame, and lack of intrinsic motivation.^18^ Unique enablers include perceived health benefits such as improved disease control, continuation of an active lifestyle and normal functioning, receiving guidance from health professionals, and program-related factors such as social interaction.^17^–^19^

Most of the previous research has focused on PA as part of PR and on individuals with COPD from secondary care settings. However, to date there is a lack of qualitative studies exploring the attitudes and beliefs of individuals with COPD from the full spectrum of disease severity and with the specific focus on initiating and maintaining daily PA as a lifestyle choice, rather than as part of a structured psycho-educational program,^18^,^19^ something that would be useful given many individuals with COPD are managed in a primary care setting.^20^

## Aims and objectives

The primary aim of this study was to examine the barriers to and enablers of PA in individuals with the full range of COPD severity in a primary care setting. Although the majority of past research has examined PA as part of a structured PR program,^18^ to date, there has been little attention to differentiating between PA as part of PR and PA as part of daily life. Due to PR not being accessible for everyone in the community^19^ (eg, lack of transportation, cost, duration of PR), it is important to identify barriers and enablers relevant to home-based daily PA. It is likely that incorporating PA as part of a healthy lifestyle would relate to more internal barriers/enablers compared to the program-specific barriers/enablers of PA experienced in PR. Therefore, the aim of this research is to get a better understanding of the unique factors that impact PA at home as part of a daily routine. Improved understanding will be used to inform future interventions. Social cognitive theory (SCT),^21^ a widely applied theory to explain the complexity of behavior change, was the framework used to interpret the results. Constructs of this framework (eg, self-efficacy, perceived barriers, outcome expectations, and self-regulatory behavior) have been shown to explain almost a third of the variance in PA behavior in healthy populations.^22^

## Methods

### Participants

Participants were drawn from the Birmingham COPD Cohort Study,^23^ which includes individuals with established COPD on primary care disease registers and case-found patients identified through a program of COPD screening. A purposive sampling strategy was used to select men and women with both established disease and those who were case found.^24^ We sought to include individuals from a wide age range and disease severity and within this to specifically invite individuals previously referred to PR and those in full-time employment.

### Procedure

Study invitation letters were sent to 404 patients from the Birmingham COPD Cohort Study, with one reminder for non-respondents. Invitees were selected based on their proximity to the research center and to represent the range of characteristics sought. From those invited, 79 individuals expressed an interest in participating, and individuals were contacted based on their characteristics and time availability to join one of four predefined focus groups. All participants provided written informed consent. To optimize our understanding of PA behavior relevant to the COPD experience, extra care was given to create distinct groups that represented different characteristics. These groups were created with the aim to acquire a greater depth of perceptions and not for the purpose of comparing different groups. To allow for a more detailed evaluation, group differences will be explored within a further publication. The groups included patients who 1) were recently case found, 2) reported previous referral to PR, and 3) were in current paid employment and 4) a mixed group, which was heterogeneous in terms of gender, age, and severity. We aimed to recruit five to eight participants per group.^25^ Within each group, we tried to ensure an equal allocation of participants in terms of age, gender, and breathlessness. This process was carried out while taking into account the potential influence of contextual factors on PA engagement in individuals with COPD (eg, employment/previous experience of exercise as part of PR or any other structured program) to allow for a range of perspectives to emerge. Focus groups took place in a university setting during July 2014. Three of the researchers (MCK, NH, CR) conducted the focus groups with interchangeable roles, moderator and observer. Focus groups were audio recorded, with an average duration of ~90 minutes.

### Interview guide

A semi-structured interview guide^26^ was informed by SCT, with each question aimed at targeting its major components. The interview guide had been piloted with healthy older adults in a prior study^14^ and was modified to reflect aims and population (eg, wording changes, provision of COPD-specific examples) for this study. Questions explored the perceived benefits of PA, views on the importance of setting PA goals, ways of incorporating PA into one’s daily routine, obstacles faced when engaging in PA, and factors that made it easier to be active. Probes were used to extract more in-depth information (eg, “Help me to understand what you mean?”).

### Data analysis

Focus group discussions were transcribed verbatim. Thematic analysis^27^ was undertaken by MCK in discussion with NH, CR, SEW, and JC. Quotations, referenced by participant grouping and Medical Research Council (MRC) score, were selected to illustrate themes and sub-themes and to ensure that the findings represent the participants’ voices. As a method of triangulation of evidence, example quotes were presented and discussed in the group meetings with the primary investigators until consensus was reached on the coding frame. As no new themes emerged in the final group, data saturation was assumed.^28^ Rather than using a preexisting coding framework,^27^ the data were inductively analyzed to allow for new findings to emerge that did not necessarily fit within SCT. Data trustworthiness was maximized by reviewing and discussion of coding among the research team, reflection on random quotes, and researcher reflexivity.

### Ethics

Approval to conduct this study was obtained from the National Research Ethics Service (REC reference: 11/WM/0304).

## Results

### Participant characteristics

Twenty-six participants (15 male, 11 female) aged between 50 and 89 years took part in the four focus groups. A total of 42.3% of the participants had moderate-to-severe breathlessness (MRC grades 3–5) while they represented a range of severity in terms of airflow obstruction. Table 1 shows the distribution of participants in each focus group. Although overall there was a balance of participant characteristics across the different groups, there were some clear differences in the makeup of each of the focus groups. For instance, Group 1 is composed of younger and less impaired individuals compared to Group 4.

### Definitions of PA

Participants were asked to give a personally meaningful definition of PA, which encouraged them to reflect on their own understanding and experiences. The majority of the participants broadly defined PA as any lifestyle activity including walking, gardening, and housework, as part of their daily routine. For instance, one participant explained,

PA is anything that keeps me bending up and down, walking along, anything that makes me feel that I’m stretching my body in some way [Employed/MRC 2].

Although not a question that specifically guided the analysis, this personalized PA definition provides some context to the responses provided by the participants. After getting a sense of the personal meaning of PA, all the participants agreed to a common definition describing PA as “any activity with the goal of achieving fitness and health while it involves other lifestyle activities”.

### Barriers and enablers to PA

**Figure 1:**
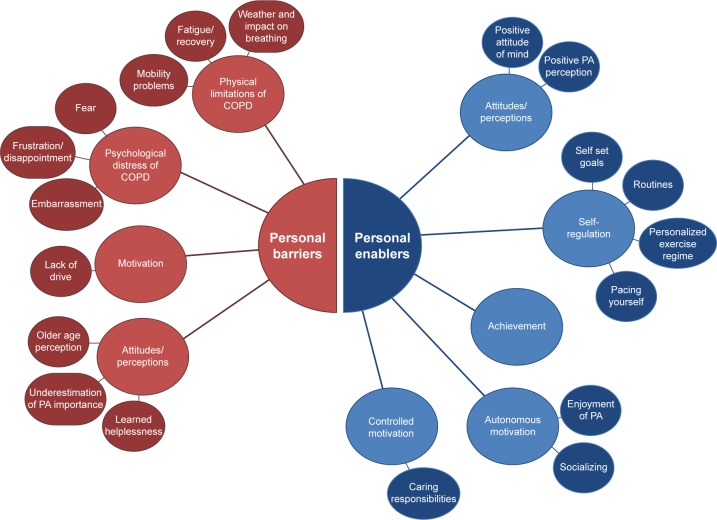
Personal barriers and enablers. **Abbreviation:** PA, physical activity.

**Figure 2:**
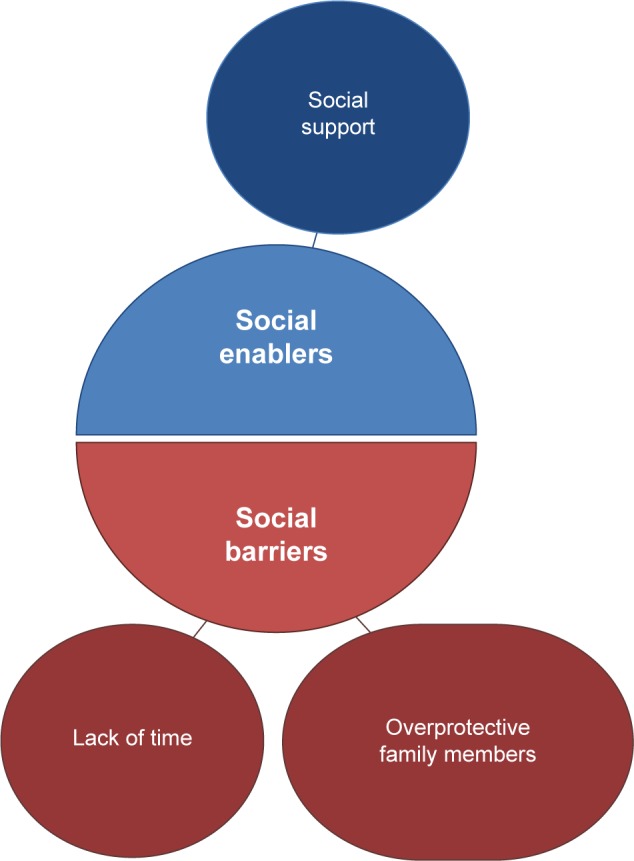
Social barriers and enablers.

The analysis identified two overall higher-order 1st level themes associated with PA: “barriers” (factors preventing engagement in PA) and “enablers” (factors encouraging engagement in PA). Within both higher-order themes, emerging 2nd level subthemes were defined as either personal (related to the individual) or social (external influences). Figures 1 and 2 summarize the resulting themes.

#### Personal barriers

Four 3rd level subthemes were identified, including physical limitations, psychological distress, lack of motivation, and negative attitudes/perceptions in relation to PA. Frequently, the participants discussed their health issues related to having COPD, including fatigue/recovery from breathlessness, mobility problems, and potential impact of weather conditions on breathing. Some participants felt unable to carry out PA as part of their daily routine due to a lack of stamina, stating, “So I’d like to be able to be more active but I haven’t got the energy basically” [Employed/MRC 3]. Other participants focused on recovery time after PA,

If you look at an athlete who’s just run, they’re out of breath but they recover much quicker, that’s the difference, their recovery time is much quicker than ours [PR/MRC 5].

Also, many participants discussed breathlessness following activity, particularly if involving an incline or lifting, and how this affected their mobility. One participant described, “I do the kitchen, clean down the woodwork and when you bend down and get up you’re short of breath” [Mixed/MRC 4]. Furthermore, adverse weather conditions (eg, wet, hot, cold, and damp air) were a major concern as they affected participants’ breathing. One participant highlighted the issue,

That affects me quite a lot, it makes me a lot more breathless and I seem to get more pain as well in my chest with the cold weather [Employed/MRC 3].

In addition to their health issues, the majority of participants talked about the psychological impact of the physical limitations associated with their COPD, including embarrassment, fear, and frustration/disappointment. In some cases, participants felt ashamed, uncomfortable, or embarrassed when experiencing symptoms such as breathlessness and immobility in front of others, along the lines of

They see you out of breath or you’re on a rollator, people do come up to you and say are you all right. You do get kind of embarrassed because you’re only taking a breather you know but they just see you’re in distress [PR/MRC 4].

Some participants reported concern when experiencing symptoms such as breathlessness or pain. One participant indicated how this felt,

When I first got the pains when I went up steps or walking up a hill, it used to actually frighten me a bit, I used to think oh god this is my heart or you know [Employed/MRC3].

Another participant while describing why PA was limited, commented,

I get panic attacks. They hit me at any time during the day; they are a growing influence on my life. I become desperate to try to move something off my chest that won’t go. I’m fighting for breath [PR/MRC 3].

A few reported experiencing negative feelings and a lack of motivation to exercise due to the irreversible nature of their disease. A participant reported, “I find [it] frustrating that I’m not able to do what I want to do” [Mixed/MRC 2]. Participants’ perception of their condition and ability to alter it defined their engagement in PA. A few participants spoke about not being in control of their condition. This feeling of being unable to change their situation and learned helplessness led them to believe that there was no point of engaging in PA. For instance, a participant said,

If your lungs are scarred, they can do nothing about that, so it’s a state of mind so you think well it’s done now, it’s too late, you know, I’m not going to bother, my lungs are getting no better, but you won’t get better so there’s no big incentive [PR/MRC 4].

A number of participants perceived PA unnecessary, justifying their lack of activity by comparing themselves to others who, although they exercised, were less healthy than themselves. For example, a participant said,

I don’t think they’re any fitter. I have got a large circle of friends and a lot of them go to the gym. One is mad keen cyclist, he goes for miles and miles and as I say I don’t do anything, but I’m the only one that don’t take any pills, I am in better condition than they are [recently case found/MRC 1].

Some participants perceived that exercise is not necessary in older age and supported their argument with the belief of not wanting to overdo it as you get older. One participant commented,

So why do we need to do all this exercising at our age now, I don’t really think you can get a massive amount of benefit at our age. Younger people yes, but our age no [recently case found/MRC 1].

Several participants discussed having no motivation to engage in PA. For instance, a participant described,

I can’t motivate myself to do physical exercise. I’ve done it when I was younger, yoga and things like that but now, I just don’t want to [recently case found/MRC 2].

#### Social barriers

This category consisted of two subthemes, overprotective family members and lack of time. A few participants discussed having family members who do everything for them, with the constant offer of help discouraging them from engaging in PA,

My husband takes me everywhere and he does everything, he won’t let me do anything though […] It is a problem when somebody is over-protective [PR/MRC 4].

Several other participants, mainly those in employment, perceived a lack of time to engage in exercise, reporting, “If I didn’t work so many hours then I’d have more hours to put aside for activity” [Employed/MRC 3].

#### Personal enablers

Five 3rd level subthemes were identified, each consisting of further subthemes: autonomous motivation, controlled motivation, attitudes/perceptions, self-regulation, and achievement. Participants described pursuing PA because of personal interests and values. For instance, one of the most frequently reported reasons for engaging in PA was that it was pleasurable. A participant said, “While I’m doing it I’m in heaven, I’m on another planet, it makes you relax” [Employed/MRC 1]. An equally frequent reason for pursuing PA was the importance of meeting other people through engagement in PA. A participant stated,

I’m motivated by the social side, the people I meet with or play. I’ll play any sport just for the social side of it; yeah I do enjoy that, that’s a motivation [Employed/MRC 1].

However, many participants reported engaging in PA due to ongoing obligations such as having to take care of another family member or a pet. Thus, external influences compelled them to be active along the lines of,

If it was just me on my own sitting there with the dog, the dog wouldn’t go anywhere, end of story. So there’s an element of that as well [PR/MRC 3].

At the same time people’s opinions of COPD and how it affects them fundamentally influenced their behavior and therefore their ability to engage in PA. A few participants recognized the importance of normalizing their condition and aiming for a regular life. Keeping a positive attitude about their condition helped them cope with related stresses and strains resulting in a brighter outlook on life, along the lines of, “Looking on the bright side of life and having a sense of humour” [Mixed/MRC2]. Another participant tried to downgrade the magnitude of the problem and disregard COPD symptoms, to the point that he denied his condition, “It never affects me; I might as well not have COPD because I don’t think I’ve got it” [PR/MRC 2]. Thus, denying their lung problems seemed to be a defense mechanism to cope with their disease.

The belief that PA can positively affect COPD appeared to be a motivational factor,

I think if you’re improving your cardiovascular health overall PA is bound to help you to manage the symptoms of COPD [recently case found/MRC 1].

Several self-regulatory strategies were employed by participants to incorporate PA into their daily lives while adjusting to the nature of their condition. The majority of participants described setting their own goals for PA, which motivated them and provided an incentive. For example, a participant stated,

I have the idea in my mind if it’s less than two miles or up to two miles I will walk rather than take the car or the bike [recently case found/MRC 1].

Another important strategy for many of the participants was establishing a habitual behavior. A participant described,

Over the years I got in the habit of getting out of bed at 6 o’clock in the morning, going downstairs, making me and my wife a cup of tea and taking it back again and then going down and repeating the exercise. So that’s sort of a very early morning routine which is something I don’t think I can give up because it’s part of my life which has to be done [PR/MRC 3].

As a way to facilitate initiation of PA and incorporating exercise into their daily lives, many participants recognized their individuality and identified an activity that they felt comfortable doing. For instance,

I use my weights. Because I’m not walking, I’m not moving, I can do my exercises and I feel comfortable with that; I do it every day [PR/MRC 4].

Other participants adopted a slower pace when engaging in PA allowing them to continue their activities, while managing their symptoms,

I do most of things that I used to do but I find that I do have to keep stopping because I get breathless with all the house-work and things […] I do try to do most things that I do just at a slower pace sometimes [Employed/MRC 1].

The majority of participants experienced feelings of satisfaction and fulfillment whenever they were able to set a PA goal and achieve it. Such positive reinforcement was an important facilitator, motivating them to maintain PA,

I’ll go somewhere where there might be a bit of walking in it as well and I feel as though I’ve achieved something, which is great [Employed/MRC 1].

#### Social enablers

Social support, referring to social and environmental factors that are somewhat beyond an individual’s control, was discussed as an important enabler of PA behavior. Feeling understood and encouraged to be physically active by partners, friends, and people with similar issues motivated some to become more active. For instance, a participant reported,

I also think that support from family, ie, your partner, husband, or wife, is pretty important on a day-to day routine. My wife might say to me, nice day out, so you fancy taking the dog for a walk in the park […] there’s a backing there [PR/MRC 3].

## Discussion

This is the first qualitative study underpinned by SCT to focus on the barriers and enablers to PA in individuals with the full range of COPD severity in a primary care setting. Our study expands on previous research^18^,^19^ by examining the reasons that motivate and hinder individuals with COPD to engage in daily PA and provides a new insight into how several contextual factors (eg, different degrees of breathlessness, onset of disease, employment status) can influence PA engagement.

Despite some similarities with previous research, our study differs in several respects from the existing literature (Table 2). For instance, Hartman et al^18^ recruited a large heterogeneous sample from a secondary care population, while our study focused exclusively on the primary care setting that contains a broader range of COPD patients. Furthermore, the description of data collection and analysis was limited in the study of Hartman et al, with the interviews not audiotaped and transcribed verbatim, raising concerns with respect to trustworthiness and accuracy of the interview data.

A more recent study by Thorpe et al^19^ examined the barriers to PA 2 months following a hospital admission. The participants had severe COPD, and the emphasis was on PR rather than PA during daily life. While recent reviews have investigated the reasons for poor adherence or dropout of PR, this has not until now been extended to include participation in PA as part of daily life.^29^,^30^ One study that did focus on the barriers specific to activities of daily living was a cross-sectional study from Brazil.^31^ The study demonstrated an association between social influences, lack of infrastructure, and lack of willpower and lower PA among people with COPD. Table 2 includes a detailed comparison of findings from previous studies on the barriers and enablers of PA in individuals with COPD.

### Barriers

The majority of barriers were related to the nature of the disease. A key barrier that emerged in this study was physical limitations and constrained mobility. Consistent with previous studies, our findings indicate the importance of mobility for participation in PA. Specifically, many participants reported difficulty with certain types of activities such as those conducted on an incline (eg, walking up the stairs). Our findings support previous research indicating that COPD-related symptoms are a barrier to patients participating in PA^19^ and that poor weather has a significant impact on breathlessness and health status.^18^

Although most research in patients with COPD has focused on environmental/situational barriers out of an individual’s control, that are relevant to the PR program^18^ (eg, PR program intensity, length of PR, transport, parking issues, financial difficulties, weather, PR setting), our study focused on more internally perceived barriers that are within an individual’s control and relate to daily life PA (eg, emotions, attitudes/perceptions, motivation). Novel data emerging from this study include the psychological and emotional impact of COPD, with participants expressing embarrassment, frustration, and disappointment at their inability to engage in PA. Existing research on the psychological distress associated with COPD is limited and focuses mostly on clinical symptoms such as depression.^32^ Furthermore, fear of breathlessness emerged as one of the main reasons for low PA levels among our sample, which may reflect participants’ lack of confidence in managing breathing difficulties. These findings are in line with previous research, which has indicated shame^18^ and fear^18^,^33^ to be related to low levels of PA in individuals with COPD. Our findings support the rationale for developing personalized PA regimes, commencing at a level that is comfortable for the individual and gradually increasing in intensity/duration.

Another unique barrier that emerged in this study was related to low outcome expectations and disregard of the potential benefits of PA. These attitudes may be related to fear of breathlessness and a lack of confidence in the ability to be active. Such attitudes were reinforced by negative self-imposed beliefs of being too old to exercise and perceived lack of control with feelings of resignation to having COPD. Whereas past research has pointed out that negative outcome expectations (eg, fatigue, exacerbation of COPD symptoms) can discourage individuals with COPD from engaging in PA,^18^ this study revealed that individuals with COPD tend to undermine the importance of PA as a way of improving their condition. The negative perception of PA along with their feeling of being helpless and not being able to control their condition can affect their outcome expectations. Health practitioners may usefully consider individuals’ beliefs and explore ways to assist them to realize the positive impact of PA on their disease.

### Enablers

We found that attitudes toward COPD, perceptions of disease impact, and beliefs that PA is beneficial appear to be important determinants of engagement in PA in this primary care population. Our finding that enjoyment promotes PA is in line with previous research, highlighting the importance of intrinsic motivation in facilitating PA.^34^,^35^ Controlled motivation (eg, PA as part of taking care of a family member) was also expressed as a stimulus to being active, although previous research suggests that this is associated with less positive psychological well-being and lower psychological need satisfaction compared to autonomous motivation.^34^ Thus, identifying and promoting pleasurable activities are important in encouraging individuals with COPD to become more physically active.

A unique feature of this study is that the participants described a range of self-regulatory strategies to manage their symptoms and motivate themselves to remain physically active (eg, keeping to a routine, having a personalized regime, and adopting a slower pace), whereas past research has primarily focused on goal setting as a way of regaining control of their condition.^18^ Although the participants in our study recognized PA as a method of achieving the goals they set for themselves, this was not their only coping strategy. Thus, self-regulating their behavior overall emerged as an important predictor of PA engagement.

The feeling of accomplishment gained from engaging in PA is an important source of self-efficacy^21^ for individuals with COPD who appeared to use PA as a mechanism to cope with the feeling of lack of control. Because of the irreversible nature of COPD, this finding suggests that individuals can benefit from successful experiences with PA. Past research has also identified sense of achievement as a personal attribute associated with higher levels of engagement in PA as part of PR. However, in our study, the sense of achievement derived from being able to achieve a personally meaningful goal and not as an outcome of participating in PR. Another important social cognitive factor impacting on PA levels was the social aspect associated with some activities.^21^ Socializing with other people seems to have a beneficial impact on PA engagement in individuals with COPD who might feel socially isolated.^36^ This finding is consistent with previous research that indicates the importance of emotional support and company as facilitators for PA engagement.^18^

### Strengths and limitations

This study is one of the few to examine the factors that inhibit and facilitate engagement in PA in a primary care COPD population underpinned by SCT. The qualitative approach allows for an in-depth understanding of the beliefs that individuals with COPD hold when it comes to changing their health behavior. While previous studies focused on external barriers such as lack of time, lack of transportation, or issues specific to interventions such as PR,^19^,^29^,^30^ we identified personal barriers related to perceptions, motivation, and attitudes toward PA. Developing future interventions according to a more holistic understanding of barriers and enablers of engagement in PA could increase attendance and adherence rates among COPD patients.

Creating distinct groups was another unique methodological feature of this study, which allowed several themes to emerge associated with specific contextual factors (eg, degree of breathlessness, onset of disease, employment status). Given the complexity of COPD and the importance of engagement in PA in patients with COPD, it is important to identify context-specific barriers, eg, working and design interventions that can enable these individuals overcome these barriers.

With the majority of participants having mild to moderate COPD, these findings may not reflect the views of those with more severe disease. Caution should also be used when interpreting the data, as it is possible that this study attracted a unique population with certain characteristics. As participants were recruited by invitation, accounting for self-selection bias is important. For instance, it is possible that people who did not volunteer to participate had no interest in PA or had more severe symptoms and mobility problems that interfered with their PA levels. As the topic guide of this study was based on the SCT framework, it is possible that the participants responded in ways supporting SCT. It is possible that using a different theoretical approach may have led to the emergence of different themes.

## Conclusion and recommendations

The results are in accordance with SCT and emphasize the importance of self-efficacy in achieving health behavior change and suggest that improving a patient’s adherence to PA can happen by building up the participant’s confidence. Those who feel confident in their ability to manage their symptoms and overcome barriers associated with their condition are more likely to self-regulate their behavior so as to incorporate PA into their daily lives. Moreover, the perceptions that individuals with COPD hold about the importance of incorporating PA into their lives and the benefits associated with it can influence PA participation. Individuals with COPD are more likely to engage in PA when they expect more positive outcomes from PA, and they believe it will improve disease management.

Our results also confirm self-regulation as one of the most influential social cognitive variables for changing health behavior.^21^ Accounting for individual differences in self-regulation is an important consideration when designing PA interventions. Thus, the findings suggest that health practitioners should help individuals with COPD to set their own personal goals, establish a daily routine, engage in personalized PA that recognizes their individual needs, and develop their own pace to complete PA.

Using SCT as a theoretical framework to understand the reasons that prevent or encourage individuals with COPD engage in PA can help in the development of theory-based interventions with the aim of promoting PA in individuals with COPD. These interventions need to be carefully designed to address all the factors that can hinder their participation while at the same time focusing on the factors that facilitate PA engagement. Health practitioners could support individuals with COPD to reframe negative cognitions related to PA participation and help them to recognize the important role played by PA as a self-management strategy and the associated benefits. Accounting for individual differences in self-regulation is also an important consideration when designing PA interventions. Thus, practitioners could help individuals with COPD to set their own personal goals, establish a daily routine, engage in personalized PA that recognize their individual needs, and develop their own pace to complete PA.

Overall, future interventions should aim to improve self-efficacy, cultivate positive outcome expectations, and support COPD patients to adjust and acquire realistic expectations regarding PA. Furthermore, as general practitioners are the first point of contact for patients with COPD, their role in exploring patients’ views on COPD and the importance of PA is crucial.

## Figures and Tables

**Table 1:** Participants’ characteristics

Characteristics	Group 1 (Employed)	Group 2 (Recently diagnosed)	Group 3 (PR)	Group 4 (Mixed)
Age group, years				
50–59	2	0	1	0
60–69	3	4	5	2
70–79	0	2	1	4
80–89	0	0	1	1
Sex				
F	3	3	2	2
M	2	3	6	5
MRC dyspnea score				
MRC1	3	2	2	0
MRC2	1	3	1	3
MRC3	1	1	3	2
MRC4	0	0	1	2
MRC5	0	0	1	0

**Notes:** MRC1: Not troubled by breathlessness except on strenuous exercise; MRC2: Short of breath when hurrying or walking up a slight hill; MRC3: Walks slower than contemporaries on level ground because of breathlessness or has to stop for breath when walking at own pace; MRC4: Stops for breath after walking ~100 m or after a few minutes on level ground; MRC5: Too breathless to leave the house, or breathless when dressing or undressing.

**Abbreviations:** PR, pulmonary rehabilitation; F, female; M, male; MRC, Medical Research Council.

**Table 2:** Summary of previous research studies: barriers and enablers to physical activity in patients with COPD

Study	Aim/question	Sample population characteristics	Design and methods	Results: main barriers identified	Results: main enablers identified	Conclusion and themes of study
Thorpe et al^19^	To identify barriers and enablers of PA 2 months after hospital admission in people with COPD	• 28 people (male =22) • Mean age =71.9 years Mild (n=2) Moderate (n=5) Severe (n=21) • Average length of stay in hospital: 6.61 days • Australian sample	• Qualitative • 28 semi-structured telephone interviews • Content analysis	Three main barriers identified with relevant subcategories: 1. The self: a. Advancing age b. Lack of access to oxygen therapy c. Negative experiences of PR (eg, lack of follow-up/intensity) 2. Health: a. Comorbidities b. Physical injury or sickness (eg, back pain) 3. Environment: a. Surroundings (eg, effect of weather) b. Transport/finance	Three main enablers identified with relevant subcategories: 1. Social: a. Support (eg, family/friends) b. Keep to a routine (ADL, eg, shopping and errands) c. Extracurricular activities (eg, hobbies) 2. Personal (individual centric): a. Feeling better (eg, positive outcomes from participation in PA and PR) b. Goal setting and self-motivation 3. Access (eg, equipment and health professionals)	Conclusion: importance of individualized targeted approach Most commonly reported barrier: poor health Further research needed on enabling strategies for participation in PA Limitation: limited generalizability
Amorim et al^31^	To evaluate the ability of COPD patients to perform ADL, to identify barriers that prevent these individuals from performing ADL, and to examine the association between activity levels and barriers	COPD group: 40 patients treated at a pulmonary outpatient clinic (male =22) Mean age =64.4 years Control group (spouses of the COPD patients and other individuals treated at the clinic): 40 healthy elderly individuals (male =19) Mean age =66.7 years • Brazilian sample	• Cross-sectional study • mMRC to determine severity of baseline dyspnea • Triaxial accelerometer to measure PA • Questionnaire to assess PA barriers (based on US CDC recommendations) • LCADL scale • 6MWT	Barriers identified: 1. Lack of time 2. Social influences 3. Lack of energy 4. Lack of willpower 5. Fear of injury 6. Lack of ability 7. Lack of infrastructure	Enablers are not actively identified in this study … But Suggestions offered to overcome the identified barriers: • Greater encouragement • More resources • Education among family members regarding disability	Conclusion: patients with COPD are less active than healthy adults Most commonly reported barriers: lack of infrastructure, lack of willpower, and social influences
Hartman et al^18^	What are the perceived reasons for people with COPD to be physically active or sedentary? Are those reasons related to the actual measured level of PA?	• 115 people (male =78) • Mean age =65 years • Mild to very severe COPD • Dutch sample • Exclusion criteria: serious active diseases or treated for an exacerbation during previous 2 months	• Observational study with qualitative element • Semi-structured interviews • Triaxial accelerometer (to measure PA) • Spirometry • mMRC (to assess dyspnea severity) • Two 6MWTs • Inductive content analysis	Four perceived reasons not to be physically active (barriers to improved health): 1. Poor weather (reported by 48% of participants) 2. Health problems (43%) 3. Lack of intrinsic motivation (11%) 4. Positive social support (50%)	Four perceived reasons to be physically active (enabling improved health): 1. Health benefits (reported by 65% of participants) 2. Enjoyment (44%) 3. Continuation of an active lifestyle in the past (28%) 4. Functional reasons (26%)	Conclusion: importance of increasing self-efficacy and tailoring the type of activity instead of a standardized PA program Themes: consistent with previous studies; enjoyment as motivation for PA is a key new theme in this study
Mathar et al^29^	To explore reasons why patients with COPD decline PR	Seven qualitative studies included in review (two from Australia, five from UK) Inclusion criteria: • Qualitative studies published in English • Studies with a patient perspective Exclusion criteria: • Reviews • Intervention studies • Lack of patient perspectives • Qualitative with high dropout or non-completion	• Metasynthesis of previous qualitative studies • Metaethnography • Five studies face-to-face interview • One study telephone interview • One mixed study (both face-to-face interview and telephone interview)	Four barriers identified in patients declining rehabilitation: 1. The referral process: a. Attitude of health care professional b. Lack of information about the process of referral and follow-up 2. Transportation and its financial burden (eg, no car or inability to use public transport) 3. Perception of health and self (eg, feelings of being too old or ill to participate) 4. Other obligations or priorities (eg, care-giving, pet care, or afraid to leave house unattended)	N/A to paper	Although: similar themes identified among the seven studies However: different aim of the studies and varied definitions Conclusion: make PR more accessible: a. At more flexible times b. In patients’ homes c. Transportation Most commonly reported barriers: the referral process, transport problems, and self-perceptions
Thorpe et al^17^	To identify potential barriers and enablers, associated with their participation in PA programs, including PR	Systematic review based on 11 studies (eight qualitative, three quantitative)	• Reviewers independently evaluated the methodological quality of the chosen papers using the McMaster critical appraisal tool	Six barriers to participation in PA and PR programs: 1. Personal issues (eg, anxiety/fear about exercise) 2. Changing health status (eg, comorbid conditions) 3. External factors (eg, weather/finance) 4. Lack of support (motivational) 5. Program related (the influence of medical practitioners) 6. Smoking (studies showed that smokers were less likely to have high attendance at PR)	Seven enablers for participation in PA and PR programs: 1. Personal drivers (self-motivation) 2. Personal attributes and perceived health benefits (persistence and positivity) 3. Social support (network formed through PR) 4. Control of condition (goal setting) 5. Program-centric enablers (safe environment and quality care) 6. Professional support (before, during, and after treatment) 7. Goal setting	Conclusion: identifies an evidence-practice gap in delivering treatment: good evidence to support the health benefits of PR, but poor implementation of PR in clinical practice. Themes: motivation and goal setting as incremental enablers
Sohanpal et al^30^	To explore factors affecting patient participation in COPD support programs	• Thematic framework synthesis on 10 of 12 studies • Studies included from 1984 to 2015	• Qualitative synthesis Theoretical frameworks used: a. “Attitude-social influence-external barriers” model b. “Self-regulation” model	Three main barriers and enablers: 1. Attitude (perception of the program as positive or negative) 2. Social influences (the positive or negative influence of the health professionals, friends, and family members in participation) 3. Intervention representations (a positive or negative perception of the support program on offer)	All three themes can be considered a barrier or enabler dependent on its positive or negative perception/experience	Conclusion: patient participation in programs is largely influenced by the participant’s attitude and social influences they are subjected to

**Abbreviations:** PA, physical activity; PR, pulmonary rehabilitation; ADL, activities of daily living; mMRC, modified Medical Research Council; LCADL, London Chest Activity of Daily Living; 6MWT, 6-minute walk test; N/A, not applicable.
